# Hepatobiliary scintigraphy allows the evaluation of short-term functional toxicity of liver stereotactic body radiotherapy: Results of a pilot study

**DOI:** 10.1371/journal.pone.0204013

**Published:** 2018-10-10

**Authors:** Berardino De Bari, Thomas Breuneval, Michele Zeverino, Sarah Godin, Letizia Deantonio, Christine Geldhof, Jean Bourhis, Nicklaus Schaefer, Raphaël Moeckli, John Prior, Mahmut Ozsahin

**Affiliations:** 1 Service de Radio-oncologie, Centre Hospitalier Universitaire Vaudois (CHUV), Lausanne, Switzerland; 2 Service de Radio-oncologie, Centre Hospitalier Régional Universitaire « Jean Minjoz », INSERM U1098 EFS/BFC, Besançon cedex, France; 3 Institut de Radiophysique, Centre Hospitalier Universitaire Vaudois (CHUV), Lausanne, Switzerland; 4 University Hospital Maggiore della Carità, Dept. of Radiation Oncology, Novara, Italy; 5 Service de Médecine Nucléaire, Centre Hospitalier Universitaire Vaudois (CHUV), Lausanne, Switzerland; North Shore Long Island Jewish Health System, UNITED STATES

## Abstract

**Purpose:**

To study the potential of (99m)Tc-Mebrofenin hepatobiliary scintigraphy (HBS) in identifying the short-term variations of liver function after stereotactic body radiotherapy (SBRT) for liver cancers.

**Material and methods:**

We treated with SBRT 3 patients (pts) affected by a cholangiocarcinoma and 3 patient presenting liver metastases (3x15 Gy, 4 pts; 5x8 Gy, 1 pt; 6x5 Gy, 1 pt). All patients received HBS before and 3 months after SBRT, which were co-registered with the simulation CT-scan. Structures corresponding to isodoses from 10–90 Gy were created, with intervals of 10 Gy. Finally, the variations of the mean activity (MBq) in each isodose structure have been calculated. Then, a linear regression analysis was performed.

**Results:**

We showed a linear reduction of the activity, significantly related to the delivered dose (p<0.01), and a reduction of the perfusion of 0.78% for each delivered Gy. The linear equation has predictive value of the loss of the function of 96% (R2 = 0.9605).

**Conclusions:**

HBS could improve treatment plans for liver SBRT, by allowing the identification of the liver function variations after SBRT and, potentially, the prediction of remnant liver function after SBRT. These preliminary results should be confirmed on long-term prospective data and larger population.

## Introduction

Stereotactic body radiotherapy (SBRT) is one of the therapeutic options in the treatment of primary or secondary liver cancers not suitable for surgery [1,2], with encouraging rates of local control (LC) and overall survival (OS) [3,4].

Radiation-induced liver disease (RILD) is a rare, but severe potential side effect of liver irradiation: several dosimetric constraints have been established and are available in the literature to reduce the risk of RILD [5,6]. Noteworthy, these constraints are based on the doses delivered on the whole liver, delineated as it appears on the simulation CT scan, but they do not take into account the regional variations of the liver function, potentially related to concomitant liver diseases (i.e., cirrhosis in patients treated for a hepatocellular cancer [HCC], hepatic toxicity of previous systemic treatments, the effects of previous local ablative therapies, etc).

99mTc-mebrofenin hepatobiliary scintigraphy (HBS) is a non-invasive nuclear medicine technique, which provides visual and quantitative information on both total and regional liver function. HBS is used on a regular basis in the preoperative assessment of future remnant liver function, follow-up after preoperative portal vein embolization, and evaluation of postoperative liver regeneration [7]. However, the integration of HBS in the optimization of radiotherapy treatment plans has been only rarely reported [8,9]. Only one case report has been published exploring the potential of HBS in monitoring the short-term variations of hepatic function (HF) after liver SBRT [10].

To the best of our knowledge, we report the first clinical experience exploring the role of HBS in the evaluation of the short-term radiation induced variations of HF after SBRT.

## Materials and methods

### HBS imaging procedure

Liver function was estimated from hepatic scintigraphy starting with a dynamic acquisition of 36 frames of 10 seconds (128 × 128-pixel matrix) starting immediately after administration of 200 MBq of 99mTc-mebrofenin (Bridatec®, GE Healthcare, Chalfont St. Giles, United Kingdom). Then, a fast SPECT/CT (Intevo®, Siemens, Erlangen, Germany) with 60 projections of 8 seconds was performed with a low-dose attenuation correction CT using iterative reconstruction (25 iterations × 4 subsets, 3D Gaussian FWHM 10 mm). Normalized hepatic clearance (in%/min/m2) was computed according the method of Ekman [11], with good reproducibility.

### Population

Between 08.2014 and 09.2015, 6 patients presented a primary (n = 3) or a metastatic liver cancer (n = 3) with an indication of SBRT. Clinical characteristics of the patients are summarized in Table 1. The total number of treated lesions was 6. For each patient, there was a clinical indication to performing a HBS, because of a clinical history of chronic liver diseases (i.e., cirrhosis, n = 3) or because of chemotherapy related toxicity before SBRT (n = 3). Table 1 summarizes data of these patients.

**Table 1:** Principal clinical and therapeutic features of the 6 patients enrolled in this analysis.

Patient n.	Treatment indication	Age at the time of the SBRT	PTV volume (cc)	Total dose/ dose fraction (Gy)	Variation of the activity after SBRT in the irradiated liver*	Other treatments in the interval between SBRT and HBS	Liver function enzymes before SBRT	Liver function enzymes post SBRT at the moment of HBS
**1**	Liver Metastases (lung cancer)	**66**	**85**	**45/15**	-17%	Chemotherapy with gemcitabine until 15 days before the SBRT Immunotherapy with Nivolumab started 15 days after the SBRT (ongoing at the moment of post treatment HBS)	AST = 25 ALT = 37 GGT = 60 ALP = 93	AST = 27 ALT = 35 GGT = 69 ALP = 100
**2**	Liver Metastases (retal cancer)	**56**	**236**	**30/5**	-1%	Chemotherapy with Folfiri + Cetuximab stopped 48 days before the SBRT and chemotherapy with Folfox + Avastin started 19 days after the SBRT	AST = 24 ALT = 33 GGT = 230 ALP = 246	AST = 23 ALT = 36 GGT = 233 ALP = 259
**3**	Liver Metastases (sigmoid cancer)	**50**	**9**	**45/15**	0%	Radiofrequency of 3 other hepatic metastases 15 days before HBS post-SBRT. No other treatments in between the 2 HBS.	AST = 230 ALT = 580 GGT = 404 ALP = 143	AST = 54 ALT = 108 GGT = 487 ALP = 208
**4**	HCC	**84**	**147**	**45/15**	-11%	Not	AST = 39 ALT = 60 GGT = 209 ALP = 203	AST = 32 ALT = 31 GGT = 184 ALP = 164
**5**	HCC	**78**	**182**	**40/8**	-1%	Not	AST = 19 ALT = 9 GGT = 466 ALP = 193	AST = 21 ALT = 12 GGT = 460 ALP = 199
**6**	HCC	82	**122**	**40/8**	0%	Not	AST = 27 ALT = 23 GGT = 46 ALP = 48	AST = 22 ALT = 19 GGT = 30 ALP = 44

Legend: HCC = Hepato-Cellular Carcinoma; PTV = Planning Target Volume; SBRT = Stereotactic Body Radiotherapy; HBS = Hepato-Biliary Scintigraphy; AST = Aspartate Aminotransferase; ALT = Alanine Transaminase; ALP = Alkaline Phosphatase; GGT = Gamma-glutamyl Transferase.

*We consider as irradiated liver, the liver receiving at least 5 Gy.

### Imaging procedures

All patients underwent a HBS before the beginning of the treatment with a mean interval of 47 days (range: 13–136). The images of the HBS were co-registered with the planning phase of the CT scan. The HBS was performed in the 13–48 days before the beginning of SBRT, except for one patient. This patient initially received a HBS in the 20 days preceding the treatment. Unfortunately, he presented a severe bowel occlusion before the beginning of SBRT, treated surgically and needing some weeks of hospitalization. When he came back to our attention to receive SBRT on the liver metastases, we considered that the HBS the he had already undergone could still be considered adequate, as he did not receive new treatments potentially changing his hepatic function.

All patients underwent a 4D, contrast enhanced simulation CT scan. In patients treated with Cyberknife (Accuray, Switzerland), 3–4 fiducials were implanted before simulation CT-scan to allow the tracking of the lesion during the treatment. The gross tumor volume (GTV) was contoured with Velocity software (Varian, USA) after deformable or rigid image registration with MRI in all the patients. In patients treated with Cyberknife and an online fiducial-based tracking, a 5-mm margin was added to the GTV to obtain the planning target volume (PTV). In patients treated with CyberKnife but without tracking, or with Tomotherapy or VMAT, an internal target volume (ITV) was defined on the 10 phases of the 4D-CT scan and a 5-mm margin was added to the ITV to obtain the PTV. The organs at risk usually contoured for a liver SBRT were delineated. Moreover, we also identified the 700 cc of the liver outside the PTV showing the higher activity at HBS, and we call this structure “healthy liver” (HL).

Then, a treatment plan was computed. Patients were treated with Cyberknife (n = 3), helical Tomotherapy (n = 2; Accuray, CH), or with volumetric modulated arc therapy (VMAT, n = 1; Elekta, UK). The usual constraints adopted in our Department for treatment plan optimization were used [12]. A further constraint was used on the HL: we established that the mean dose on this structure should be < 15 Gy. Noteworthy, it was a “secondary constraint”, as in no cases we allowed to violate the correct PTV coverage and/or the respect of the internationally accepted constraints only to allow the respect of the constraint on the HL.

**Fig 1:**
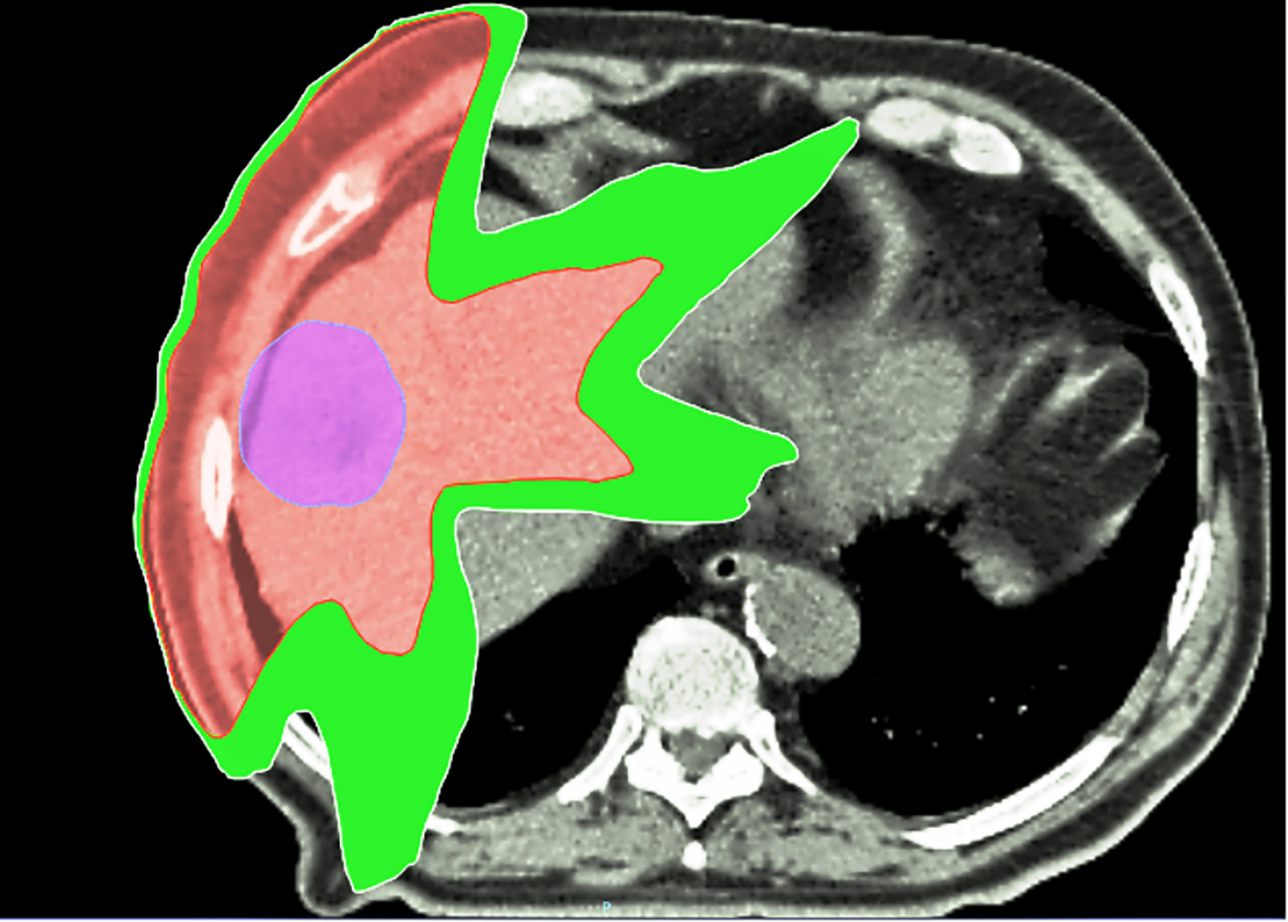
A typical image in our study: In red the 20 Gy isodoses (EQD2), in white the 10 Gy (EQD2) for a patient treated with VMAT. In green, the cropped structure (10–20 Gy crop). In this cropped structure we calculated the difference in the mean activity before and after radiotherapy. The same kind of structures and analyses were performed for all the so-obtained crop structures, calculated and defined with an interval of 10 Gy (EQD2). In violet we showed the PTV.

After a mean time of 95 days (range: 89–112), patients underwent a second HBS. Also these images were co-registered with the planning phase of the simulation CT-scan. The 2 Gy-per-fraction equivalent dose (EQD2) was calculated (alpha/beta ratio = 10 Gy for acute toxicity). On the planning phase of the simulation CT-scan, we identified the isodoses, from 10–90 Gy, with an interval of 10 Gy between each isodose (10 Gy, 20 Gy, 30 Gy, etc). Structures corresponding to the lower dose cropped with the following higher one were created. For example, considering the 10 Gy structure, we cropped the parts of the structure corresponding to 20 Gy (or more), which were inside the 10 Gy. Then, we cropped the parts of the structure corresponding to 30 Gy (or more) structure, which were inside the 20 Gy. We made the same for all the isodoses, thus obtaining several “X Gy crop” structures, which represented the interval of doses between two following considered isodoses (between 10 and 20 Gy, between 20 and 30 Gy, etc …). Fig 1 shows an example of 10 Gy isodose (in white) and 20 Gy isodose (in red) on the same CT scan image. In green, the resulting cropped structure (10–20 Gy crop). To take into account the impact of some external variables on the final liver activity (weight of the patient, injected activity, time of the image acquisition, etc), for each HBS we performed an internal normalization between the mean activity of the irradiated liver (i.e., the liver receiving at least 5 Gy) and the mean activity of the not-irradiated liver (i.e. the liver receiving less than 5 Gy). We calculated the mean normalized activity (MBq) in the each “X Gy crop” before and after the treatment (see Fig 1). Then, we calculated the difference (in%) between the pre- and post-treatment values of mean activity (deltaAct). It gave the information about the loss of HF related to the considered interval of dose.

Then a linear regression analysis (LRA) has been performed to establish if there was a predictable relation between the delivered dose and the deltaAct in each “X Gy crop”.

All patients were informed about the goal of the HBS and our analysis, and they gave their verbal consent, and it was recorded in the clinical chart of each patient. The Ethics Committee of the Swiss Vaud Canton also approved this study and the methods adopted obtain the patients consent.

## Results

**Fig 2:**
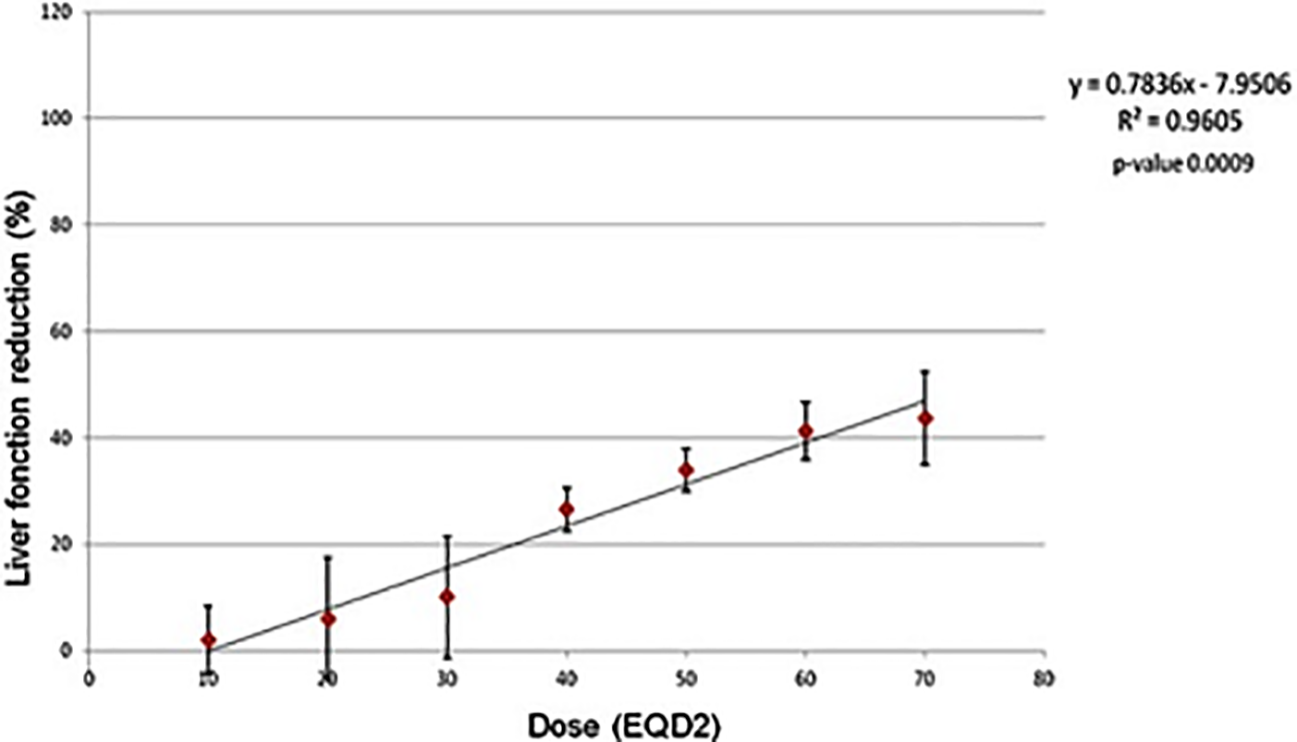
Results of the linear regression analysis.

All patient data were available for the analysis. Fig 2 shows the results of the LRA. A significant linear relation was found between the delivered dose and the loss of HF: the higher the delivered dose, the higher the loss of HF (p = 0.0009). Our analysis shows that there is mean loss of 0.78% of the HF for each delivered Gray. The predictive value of the LRA was 96%, meaning that when we adopt the obtained equation, and we replace the x with a given dose, we have 96% of possibility of predicting correctly the loss of HF at 3 months.

## Discussion

In this feasibility study we aimed at the evaluation of HBS as imaging tool in the prediction of loss of HF. Our study shows that the integration of HBS is feasible and that it could be a useful tool to identify the impact of radiotherapy on the HF. Indeed, with our approach, we showed the linar correlation between the delivered dose and the 3-month loss of HF.

Although HBS is regularly used in the pre-surgical assessment of the remnant liver function [7], its use has been only rarely reported in the radiation oncology literature. Indeed, only 2 studies have been published exploring its role in the treatment plan optimization for liver targets [8,9]. In the study by Shen et al., the authors only illustrated a procedure for the inclusion of ^99m^Tc-mebrofenin HBS for optimizing radiotherapy for primary and secondary cancers, but no clinical data are described [9].

The study by Gunvén et al. reported the potential interest of mebrofenin HBS in identifying the late side effects of liver irradiation on a population of 11 long-survivor patients affected by primary or secondary liver tumors [8]. Eight of 11 patients received a HBS after the treatment, at the earlier, 3 years after SBRT. The authors concluded that results of HBS did not relate to the irradiation. In their opinion, this lack of relationship suggests that changes due to the SBRT were too subtle and/or too short-lived to be detected. However, in their study no pre-SBRT HBS was available, so it was not possible to assess, in the post-treatment setting, any relationship between the dose and the variation of hepatic function. Moreover, most of their patients received several systemic or surgical treatments after SBRT and before HBS, a factor that could surely impact their results.

An obvious and major limitation of our study is that it only includes a mixed population of 6 liver cancer patients. The creation of a model of activity reduction on this small number of patients of varied diagnosis is not likely to be used clinically, validated or reproduced. The results of the linear regression analysis are presented only to complete the results presented in the Table 1. Indeed, although SBRT was not responsible for major loss of function in the irradiated liver, it can be found, with our approach, that in the areas of higher delivered doses the loss of function was higher, up to more than 40%, as showed in the graph of the linear regression.

In our study, we only aimed at exploring, in this very preliminary phase, the feasibility of the integration of HBS in treatment plan optimization. Clinical data that are presented could not be a final model, but only a way to show the potential of our approach, that must be confirmed in large, prospective cohorts. Moreover, some of the treatments delivered after SBRT and before the second HBS in some of the patients (see Table 1) could potentially affect the results. Nevertheless, the analysis of the hepatic blood chemistry did not show a deterioration of the liver function, meaning that these treatments did not negatively affect the results of our analysis. It should be noted that the first evaluation of the hepatic blood test has been performed 3 months after the end of RT. So, we could not know if we missed any transient variation of these parameters happening in these 3 months. In any case, none of our patients showed major variations at 3 months. Whether this result is related or not to our healthy liver-sparing approach is difficult to be established. Considering the small number of patients, we did not perform *ad hoc* histology based analyses (i.e., primary vs secondary liver cancers), but we acknowledge that it would be of clear interest, also to show different subclinical variations, which could depend on the baseline conditions of the liver. Indeed, it could be argued, for example, that a cirrhotic liver could present a different tolerance to radiation than a normal liver. We planned these kinds of analyses in a prospective trial with larger and more homogeneous populations. It should also noticed that the goal of the study was not to establish the effectiveness of SBRT in liver cancer (that could be potentially affected by the histology of the treated lesions), but to assess the potential clinical usefulness of this innovative approach.

Nevertheless, our results are hypothesis-generating, as they support the adoption of nuclear medicine and molecular imaging not only for target delineation, but also to introduce new functionally based approaches in treatment plan optimization aiming at the reduction of the treatment-related toxicities. These approaches are appealing in the modern *era* of intensity-modulated radiotherapy, as it allows the selective sparing of certain areas of interest. For example, looking at the available data, SBRT could be responsible for an early decline in the Child-Pugh score in almost 15–30% of the patients treated with SBRT for a HCC [13–16]. The integration of functional imaging allows the identification of the more functioning areas of the liver, thus allowing the possibility of selectively spare these areas. Such a kind of approach has been already successfully adopted for the treatment of thoracic targets [17]. Moreover, these function-based approaches could allow, in the future, the analysis of the effect of the same dose on different baseline level of HF. The assumption of this kind of analyses is that areas of the liver contributing lesser to the whole HF could be characterized by different radiosensitivity compared to more functioning ones, and then that the same dose could affect them differently. Then, it opens to new interesting horizons, as it could improve the optimization of the radiotherapy plans, which could be then tailored on the distribution of the individual liver function. Such kind of studies would allow the identification of predictive algorithms to calculate the remnant HF after liver SBRT, thus tailoring the treatments and the indications, and opening the possibility of treat with SBRT also patients with more limiting HF, by selecting sparing more functional areas and by defining functional based constraints, ensuring a remnant HF still compatible with life.

Noteworthy, by repeating HBS at different moments during and after SBRT, our approach allows to obtain interesting insight about the timescale of liver repair and regeneration, and then it could be an exciting tool to understand the radiobiology of liver, and deserves further, prospective evaluations.

## Conclusions

Our pilot study shows the interests of integrating hepatobiliary scintigraphy in the treatment plans of liver SBRT. This organ function-adapted approach allows the evaluation of the function impact of SBRT. We found a linear correlation between the delivered dose and the loss of hepatic function. Larger populations and long-term evaluations are warranted to confirm our data and the usefulness of optimization approaches based on the organs at risk function.
